# Associations of Increased WIC Benefits for Fruits and Vegetables With Food Security and Satisfaction by Race and Ethnicity

**DOI:** 10.5888/pcd21.230288

**Published:** 2024-03-28

**Authors:** Marisa M. Tsai, Christopher E. Anderson, Shannon E. Whaley, Catherine E. Yepez, Lorrene D. Ritchie, Lauren E. Au

**Affiliations:** 1 Nutrition Policy Institute, University of California, Division of Agriculture and Natural Resources, Oakland, California; 2 Public Health Foundation Enterprises WIC Program, Division of Research and Evaluation, City of Industry, California; 3 Department of Nutrition, University of California, Davis

## Abstract

**Introduction:**

The Special Supplemental Nutrition Program for Women, Infants, and Children (WIC) provides nutrition support for racially and ethnically diverse populations. In 2021, the monthly cash value benefit (CVB) for the purchase of fruits and vegetables increased from $9 to $35 and was later adjusted to $24. This study investigated, by racial and ethnic groups, whether CVB increases were associated with increases in CVB redemption, household food security, child fruit and vegetable intake, satisfaction with CVB amount, and likelihood of continued participation in WIC if the CVB returned to $9 per month.

**Methods:**

We conducted a longitudinal study of WIC participants (N = 1,770) in southern California at 3 time points, from April 2021 through May 2022; the CVB amount was $9 at baseline, $35 at Survey 2, and $24 at Survey 3. Racial and ethnic groups were Hispanic English-speakers, Hispanic Spanish-speakers, non-Hispanic Asian, non-Hispanic Black, non-Hispanic Other, and non-Hispanic White. We used mixed-effect and modified Poisson regressions to evaluate outcomes by group.

**Results:**

At baseline, groups differed significantly in dollars of CVB redeemed, percentage of CVB redeemed, household food security, and satisfaction with CVB amount. After the increase in CVB, we found increases in all groups in CVB redemption, household food security, and satisfaction. Non-Hispanic Black and Hispanic English-speaking groups, who had low levels of satisfaction at baseline, had larger increases in satisfaction than other groups. Reported likelihood of continued WIC participation if the monthly CVB returned to $9 also differed significantly by group, ranging from 62.5% to 90.0%.

**Conclusion:**

The increase in CVB for children receiving WIC benefited all racial and ethnic groups. Continued investment in an augmented CVB could improve health outcomes for a racially and ethnically diverse WIC population.

**Summary**

**What is already known on this topic?**

Racial and ethnic disparities in food access and dietary intake persist. The Special Supplemental Nutrition Program for Women, Infants, and Children (WIC) serves a racially and ethnically diverse population with low income. Trends related to the augmented cash value benefit (CVB) have not been assessed among racial and ethnic groups.

**What is added by this report?**

Racial and ethnic groups experienced improvements in food security, satisfaction with CVB amounts, and likelihood to continue receiving WIC but differed in baseline levels and magnitude of increases. Results highlight the importance of studying WIC participants by racial and ethnic groups to optimize program quality.

**What are the implications for public health practice?**

Benefits reported among diverse WIC participants support policies to make the augmented CVB permanent.

## Introduction

The federally funded Special Supplemental Nutrition Program for Women, Infants, and Children (WIC) provides nutritious food, nutrition education, breastfeeding support, and referrals to health and social services for infants, children, and pregnant and postpartum women from households with low income (1). The program serves a racially and ethnically diverse population: in 2018, 59% self-reported race as White, 22% as Black or African American, 9% as American Indian or Alaska Native, 6% as 2 or more races, 4% as Asian, and 1% as Native Hawaiian or Pacific Islander; 41% self-reported ethnicity as Hispanic (2).

The COVID-19 pandemic exacerbated racial and ethnic disparities in health and decreased household food security among households with children (3–5). In response, through the American Rescue Plan Act of 2021, the US Department of Agriculture temporarily increased the cash value benefit (CVB) for fruits and vegetables in the WIC food package for children aged 1 to 4 years from $9 per month per child to $35 per month per child from June through September 2021 (6,7). In October 2021, the CVB augmentation was extended and adjusted to $24 per month; in October 2022, it was further revised to $25 per month (8,9). Studies reported increases in fruit and vegetable intake, household food security, and satisfaction with the CVB amount after the CVB augmentation (10–14); however, no studies have assessed trends by race and ethnicity. Given the diverse racial and ethnic make-up of WIC participants and previously reported differences in the association between WIC program elements and outcomes among racial and ethnic groups, examining potential differences in outcomes across groups is an important consideration for program effectiveness (15–18).

The CVB for fruits and vegetables is a unique component of WIC food packages in that it maximizes flexibility in choices and allows for the food preferences of the many cultural, racial, and ethnic groups served (19). Fruit and vegetable consumption among US children falls below the recommendations of the *Dietary Guidelines for Americans, 2020–2025*, particularly among children from low-income households (20–22). Adherence to the dietary guidelines can reduce the risk of heart disease, diabetes, and obesity; these diet-related health conditions disproportionately affect racial and ethnic minority populations (21,23). An augmented CVB can reduce racial and ethnic disparities in health by increasing access to fruits and vegetables and program participation, through increased program appeal and cultural accessibility (19). Furthermore, differences in intake of fruits and vegetables, preferences for components of the WIC food package, and satisfaction with WIC services among racial and ethnic groups receiving WIC (15–18) support the need for evaluating the CVB augmentation by group. For example, a 2019 survey in California found that while the most common motivation across racial and ethnic groups for WIC participation was the fruit and vegetable component, Hispanic Spanish-speakers were more likely than other racial and ethnic groups to be satisfied with the CVB amount (16,17). The objective of this study was to investigate, by racial and ethnic group, whether the CVB augmentation was associated with increases in CVB redemption, household food security, fruit and vegetable intake, and satisfaction in CVB amounts in a racially and ethnically diverse sample of children in California receiving WIC.

## Methods

The study team conducted a prospective cohort study comprising 3 survey waves of caregivers with children aged 1 to 4½ years at baseline who were receiving WIC in 7 WIC clinics in southern California at the following 3 time points: pre-augmentation at $9 per month (Survey 1 [baseline], April–May 2021), during the 4-month increase to $35 per month (Survey 2, August–September 2021), and after the CVB was adjusted to $24 per month (Survey 3, April–May 2022). The survey population was selected to ensure that the sample included broad representation of the racially and ethnically diverse WIC population. Further detail on the 3 surveys is available elsewhere (10).

### Participants and recruitment

We selected all caregivers with age-eligible children from WIC administrative records and notified them about the survey by SMS message. Up to 6 follow-up texts were sent. Survey 2 and Survey 3 were limited to participants of the baseline survey to facilitate assessment of changes in outcomes. We entered Survey 2 and Survey 3 participants into a raffle for $50 gift cards; 20 winners were chosen at random for each survey. At the end of each survey, participants were asked for their consent to use their responses for research. The California Department of Health and Human Services Institutional Review Board approved the study.

### Instruments

Surveys were administered online in English or Spanish. To capture data on household composition and size, the survey assessed the number of children in the household receiving WIC and whether the household had children aged <18 years. The survey examined household food security, fruit and vegetable intake, satisfaction with the CVB amount, likelihood of continuing to participate in WIC, and change in amount and variety of fruits and vegetables consumed. Questions were written in English, translated into Spanish by native Spanish speakers, piloted, and revised accordingly. For each child receiving WIC, respondents reported the first 2 letters of the child’s name and their sex, year of birth, and fruit and vegetable intake. We determined CVB redemption amounts and percentage by examining data from electronic benefit transfer card transactions.

We collected self-reported data on respondent race, ethnicity, and maternal language preference from WIC administrative data. Race categories were Asian (including Indian, Cambodian, Chinese, Filipino, Hmong, Japanese, Korean, Laotian, Vietnamese, Thai), Black (including Black or African American), White, and Other (including multiple races, Fijian, Samoan, Tongan, Guamanian or Chamorro, Native Hawaiian, American Indian or Alaska Native). Ethnicity included Hispanic and non-Hispanic. We combined race, ethnicity, and language into the following categories: Hispanic English-speaking, Hispanic Spanish-speaking, non-Hispanic Asian, non-Hispanic Black, non-Hispanic Other, and non-Hispanic White. We conducted separate analyses of Hispanic groups by language because English-speaking and Spanish-speaking Hispanic participants have different levels of fruit and vegetable intake and satisfaction with WIC (18,24). 

### Outcome variables

**Redemption of CVB**. We assessed monthly CVB redemption by household in 2 ways: as a dollar amount of allotment redeemed and as percentage of total allotment redeemed. Data on redemption are captured at the household level, so households with multiple WIC participants receive higher CVB amounts than households with a single WIC participant. Redemption from May 2021, Sept 2021, and May 2022 align with Survey 1, Survey 2, and Survey 3, respectively.

**Household food security.** Household food security at each time point was assessed by using the US Department of Agriculture’s 6-item Food Security Survey Module (25). The tool is designed to capture household food security status during the previous 30 days at the household level; we dichotomized responses as food secure or food insecure according to protocol.

**Fruit and vegetable intake of child**. Fruit and vegetable intake during the previous 30 days was assessed for each child receiving WIC, at each time point, by using the National Health and Nutrition Examination Survey Dietary Screener Questionnaire (26), a validated tool to measure dietary intake among US populations. Scoring algorithms converted responses to estimated quantities of fruit and vegetable intake (in cups per day), based on age- and sex-specific 24-hour dietary recall (27). We calculated total fruit and vegetable, including legumes, fried potatoes, and 100% juice.

**Satisfaction with CVB amount.** Respondents were asked, “What do you think about the [$9, $35, or $24] amount for fruits and vegetables for children ages 1 through 4 on WIC? Would you say it is [too much, not enough, just right, don’t know]?” This question was adapted from a previous survey (24). We dichotomized these answers into satisfied (too much, just right) and not satisfied (not enough). We excluded from analyses responses of “don’t know” (<5% of responses).

**Changes in amount and variety of fruit and vegetable intake.** Only Survey 3 assessed these outcomes. Respondents were asked, “Has the increase in the fruit and vegetable benefit changed the VARIETY or NUMBER of DIFFERENT TYPES of fruits your child eats?” and “Has the increase in fruit and vegetable benefit changed the AMOUNT of fruits your child eats?” Questions for vegetables followed the same format. Because some respondents had multiple children receiving WIC, questions were asked in reference to their eldest child receiving WIC. Respondents reported whether their child ate more, the same, or less variety and a greater, the same, or a lesser amount. Another response option was “don’t know/not sure.” We dichotomized responses into increased variety or amount and did not increase variety or amount. We excluded from analysis responses of “don’t know/not sure” (<3% of responses). Questions were adapted from a previous WIC survey (28).

**Likelihood of continuing WIC.** Survey 3 respondents were asked, “If the amount you receive for fruits and vegetables went back to $9 instead of $24 per month, how likely are you to keep coming to WIC for your children between age 1-4?” Answer options were “very likely,” “somewhat likely,” “somewhat unlikely,” and “not very likely.” We dichotomized responses into likely (very likely, somewhat likely) and unlikely (somewhat unlikely, not very likely).

### Data analysis

We identified and matched individual children across surveys at each time point by using the first 2 letters of their name, their sex, and birth year. We limited the analytic sample to children with at least 1 follow-up survey completed and analyzed these data at the child level. We calculated descriptive statistics for baseline demographic characteristics for the full sample and for each racial and ethnic group. We used analysis-of-variance *F* tests and χ^2^ tests of independence to test for demographic differences between groups.

We assessed the dichotomous variables of household food security and satisfaction with CVB amount by using generalized estimating equation (GEE) modified Poisson regression models with robust SE estimation, accommodating repeated observations of individual children and clustering within families (29). We adjusted models for number of children receiving WIC in the household and the presence of 3 or more children (aged <18 y) in the household. We evaluated continuous outcomes (CVB redemption dollar amount, CVB redemption percentage, and child fruit and vegetable intake) in mixed-effects regression models accommodating repeated observations of individual children and clustering within families, and adjusted for number of children receiving WIC in the household and presence of 3 or more children (aged <18 y) in the household. The model with child fruit and vegetable intake as the outcome also adjusted for baseline fruit and vegetable intake and child sex and included random intercepts and random slope for child age.

We assessed results by racial and ethnic group in 3 ways. First, we stratified data by group and calculated descriptive statistics on the outcomes of interest at each time point. We tested differences between average values of each outcome between time points separately by group to evaluate within-group trends. If the overall *P* value for differences over time was significant at a .05 level, we assessed pairwise comparisons. Second, we tested whether a racial or ethnic group modified the effect of time on average values of outcomes by including an interaction between group and time point in regression models. Baseline data served as the reference point. We expressed estimates for dichotomous outcomes as prevalence rate ratios (PRRs) and 95% CIs and estimates for continuous outcomes as mean differences and 95% CIs. Finally, we assessed between-group differences in outcomes at each time point, using Hispanic English-speakers as the reference; we expressed dichotomous outcomes as PRRs and 95% CIs and continuous outcomes as mean differences and 95% CIs. If the overall *P* value for differences by racial and ethnic group was significant at a .05 level, we assessed pairwise comparisons.

For the questions in Survey 3 about changes in amount and variety of fruit and vegetable intake and likelihood of continuing with WIC if the CVB amount returned to $9 per month, we assessed differences by racial and ethnic group by using GEE-modified Poisson regression models with robust SE estimation, accommodating clustering within families, adjusted for number of children receiving WIC in the household and presence of 3 or more children (aged <18 y) in the household. If the overall *P* value for differences by racial and ethnic group was significant at a .05 level, we assessed pairwise comparisons. We conducted all analyses in SAS version 9.4 (SAS Institute Inc). All statistical tests were 2-sided, and *P* < .05 was considered significant.

## Results

Surveys were completed for a total of 3,000 children from 2,784 families at baseline (30% response rate). The analytic sample consisted of 1,770 children (1,578 households) with at least 1 follow-up survey completed (59% of baseline sample). Nearly half of the analytic sample were girls; mean age at Survey 1 was 2.8 years (Table 1). The largest group was Hispanic English-speakers, followed by Hispanic Spanish-speakers. The average number of children in the household aged 1 to 4 years receiving WIC was 1.3 and approximately one-third of households (35%) had 3 or more children (aged <18 y). The number of children in the household receiving WIC and number of households with 3 or more children differed across racial and ethnic groups.

**Table 1:** Baseline Characteristics of a Sample of Children Receiving WIC, Southern California, 2021–2022^a^

Characteristic	Total (N = 1,770)	Hispanic		Non-Hispanic			
		English-speaking (n = 798)	Spanish-speaking (n = 532)	Asian (n = 65)	Black (n = 222)	White (n = 39)	Other^b^ (n = 114)
Age of child, mean (SD), y	2.8 (1.1)	2.7 (1.1)	2.9 (1.1)	2.8 (1.2)	2.8 (1.0)	2.6 (1.2)	2.7 (1.1)
Female child, no. (%)	832 (47.0)	363 (45.5)	253 (47.6)	33 (50.8)	108 (48.7)	18 (46.2)	57 (50.0)
No. of children aged 1–4 y in household currently receiving WIC, mean (SD)	1.3 (0.5)	1.3 (0.5)	1.2 (0.5)	1.2 (0.4)	1.3 (0.6)	1.5 (0.6)	1.4 (0.7)
≥3 Children in household (aged <18 y), no. (%)	618 (35.0)	268 (33.7)	226 (42.6)	14 (21.5)	56 (25.2)	9 (23.1)	45 (39.8)

Abbreviation: WIC, Special Supplemental Nutrition Program for Women, Infants, and Children.

a Data source: a prospective cohort study comprising 3 survey waves of caregivers with children aged 1 to 4½ years at baseline who were receiving WIC in 7 WIC clinics in southern California at the following 3 time points: pre-augmentation at $9 per month (Survey 1, baseline, April–May 2021), during the 4-month increase to $35 per month (Survey 2, August–September 2021), and after the CVB was adjusted to $24 per month (Survey 3, April–May 2022) (10). Values may not add to total because of survey nonresponse.

b Includes multiple races, Fijian, Samoan, Tongan, Guamanian or Chamorro, Native Hawaiian, American Indian or Alaska Native.

### CVB redemption dollar amount and percentage

The mean dollar amount of CVB redeemed at baseline ranged from $11.05 among non-Hispanic Asians to $14.61 among non-Hispanic Others (Table 2). The dollar amount of CVB redeemed changed among all racial and ethnic groups between time points, with the lowest redemption amount at Survey 1 and highest redemption amount at Survey 2. We found no effect modification by group on the change in the dollar amounts redeemed over time (Table 3). At Survey 1 and Survey 2, Hispanic English- and Spanish-speakers redeemed higher dollar amounts than other groups; we found no significant differences between groups at Survey 3 (Table 4).

**Table 2:** Outcomes at Each Time Point Among a Sample of Children Participating in WIC, by Race and Ethnicity, Southern California, 2021–2022^a^

Item	Survey 1 (n = 1,770)	Survey 2 (n = 1,458)	Survey 3 (n = 927)	*P* value
**CVB redemption amount, mean (SD), US dollars^b^**				
Hispanic English-speaking	12.52 (5.59)^c^	44.84 (19.69)^d^	33.98 (19.49)^d^	<.001
Hispanic Spanish-speaking	12.18 (5.57)^c^	43.74 (18.41)^d^	33.42 (17.73)^d^	<.001
Non-Hispanic Asian	11.05 (4.71)^c^	42.74 (18.51)^d^	29.24 (12.39)^d^	<.001
Non-Hispanic Black	11.36 (5.43)^c^	40.13 (20.02)^d^	29.77 (16.03)^d^	<.001
Non-Hispanic White	14.46 (5.58)	48.76 (20.62)^d^	33.37 (18.79)^d^	<.001
Non-Hispanic Other^e^	14.61 (7.62)^c^	48.36 (26.16)^d^	40.72 (24.10)^d^	<.001
**Percentage of total CVB allotment that was redeemed, mean (SD)^b^^,^ f**				
Hispanic English-speaking	86.5 (31.6)	83.6 (28.6)	87.0 (29.2)	.09
Hispanic Spanish-speaking	90.7 (26.6)	90.7 (22.0)	94.1 (18.9)	.07
Non-Hispanic Asian	92.0 (21.4)	85.9 (28.3)	88.5 (28.2)	.17
Non-Hispanic Black	77.0 (39.2)	72.6 (37.2)	84.2 (30.3)	.09
Non-Hispanic White	87.6 (28.0)	85.0 (28.2)	82.2 (36.7)	.23
Non-Hispanic Other^e^	81.8 (35.1)	83.5 (25.5)	78.9 (33.8)	.91
**Household reported being food secure, no. (%)^g^^,^ h**				
Hispanic English-speaking	390 (48.9)^c^	409 (62.8)^d^	235 (57.6)^d^	<.001
Hispanic Spanish-speaking	234 (44.0)^c^	229 (51.1)^d^	153 (50.8)^c^	.002
Non-Hispanic Asian	31 (47.7)	30 (54.6)	19 (59.4)	.24
Non-Hispanic Black	86 (38.7)^c^	93 (51.4)^d^	43 (41.0)^c^	.001
Non-Hispanic White	13 (33.3)	18 (56.3)	8 (42.1)	.07
Non-Hispanic Other^e^	48 (42.1)	46 (50.6)	28 (45.2)	.26
**Fruit and vegetable intake, mean (SD), cups per day^b^^,^ i**				
Hispanic English-speaking	2.6 (1.0)^c^	2.5 (0.9)^d^	2.5 (0.8)^d^	<.001
Hispanic Spanish-speaking	2.4 (0.8)^c^	2.4 (0.8)^c^	2.2 (0.8)^d^	.01
Non-Hispanic Asian	2.4 (0.6)	2.3 (0.7)	2.5 (0.7)	.34
Non-Hispanic Black	2.5 (1.0)	2.5 (1.0)	2.4 (1.0)	.39
Non-Hispanic White	2.3 (0.9)	2.4 (0.8)	2.5 (0.7)	.67
Non-Hispanic Other^e^	2.4 (0.9)	2.6 (0.9)	2.4 (0.9)	.23
**Reported satisfaction with CVB amount, no. (%)^h^^,^ j**				
Hispanic English-speaking	45 (5.8)^c^	476 (79.9)^d^	153 (42.4)^d^	<.001
Hispanic Spanish-speaking	54 (11.0)^c^	288 (72.4)^d^	111 (41.9)^d^	<.001
Non-Hispanic Asian	2 (3.1)	34 (66.7)	10 (38.5)	—k
Non-Hispanic Black	17 (7.8)^c^	131 (80.9)^d^	53 (56.4)^d^	<.001
Non-Hispanic White	1 (2.6)	18 (69.2)	6 (33.3)	—k
Non-Hispanic Other^e^	12 (10.8)^c^	55 (74.3)^d^	24 (47.1)^d^	<.001

Abbreviations: CVB, cash value benefit; WIC, Special Supplemental Nutrition Program for Women, Infants, and Children.

a Data source: a prospective cohort study comprising 3 survey waves of caregivers with children aged 1 to 4½ years at baseline who were receiving WIC in 7 WIC clinics in southern California at the following 3 time points: pre-augmentation at $9 per month (Survey 1, baseline, April–May 2021), during the 4-month increase to $35 per month (Survey 2, August–September 2021), and after the CVB was adjusted to $24 per month (Survey 3, April–May 2022) (10). Values may not add to total because of survey nonresponse.

b Differences in continuous outcomes (CVB redemption amount, CVB redemption percentage, and fruit and vegetable intake) were evaluated in mixed effects regression models accommodating repeated observations of individual children and clustering within families and adjusted for number of children receiving WIC in the household and the presence of ≥3 children (aged <18 y) in the household.

c,d Values sharing a common superscripted letter are not significantly different from each other in pairwise comparisons that use a .05 level of significance.

e Includes multiple races, Fijian, Samoan, Tongan, Guamanian or Chamorro, Native Hawaiian, American Indian or Alaska Native.

f Redemption data were assessed at the household level. Households with multiple WIC participants receive higher CVB amounts than households with a single WIC child, so means at each time point exceed $9 (Survey 1), $35 (Survey 2), $24 (Survey 3).

g Responses to question on food security were dichotomized as food secure or food insecure.

h Differences in dichotomous outcomes (household food security and satisfaction with CVB amount) were evaluated in generalized estimating equation modified Poisson regression models with robust SE estimation, accommodating repeated observations of individual children and clustering within families, adjusted for number of children receiving WIC in the household and the presence of ≥3 children (aged <18 y) in the household.

i The model with fruit and vegetable intake was additionally adjusted for baseline fruit and vegetable intake and child sex and included random intercepts and random slope for child age.

j Responses were dichotomized into satisfied (too much, just right) and not satisfied (not enough).

k Statistical testing for differences in satisfaction with CVB amount among Asian and White groups did not meet required regression assumptions and were not conducted.

**Table 3:** Changes in Outcomes Among a Sample of Children Participating in WIC, by Race and Ethnicity, Southern California, 2021–2022^a^

Item	Survey 1 (n = 1,770)	Survey 2 (n = 1,458)	Survey 3 (n = 927)	*P* value^b^
**CVB redemption amount, difference (95% CI), US dollars^c^**				
Hispanic English-speaking	0 [Reference]	32.42 (31.14 to 33.71)	21.8 (20.27 to 23.33)	.17
Hispanic Spanish-speaking	0 [Reference]	31.88 (30.35 to 33.41)	22.35 (20.59 to 24.12)	
Non-Hispanic Asian	0 [Reference]	31.37 (27.03 to 35.71)	19.10 (13.73 to 24.46)	
Non-Hispanic Black	0 [Reference]	28.41 (25.82 to 30.99)	20.38 (17.26 to 23.50)	
Non-Hispanic White	0 [Reference]	34.29 (28.55 to 40.03)	20.53 (13.43 to 27.62)	
Non-Hispanic Other^d^	0 [Reference]	34.46 (31.06 to 37.87)	25.29 (21.31 to 29.27)	
**Percentage of total CVB allotment that was redemption, difference (95% CI)^c^**				
Hispanic English-speaking	0 [Reference]	−2.83 (−5.38 to −0.29)	−0.46 (−3.52 to 2.60)	.33
Hispanic Spanish-speaking	0 [Reference]	−0.31 (−3.39 to 2.78)	3.05 (−0.56 to 6.65)	
Non-Hispanic Asian	0 [Reference]	−5.96 (−14.71 to 2.78)	−4.78 (−15.69 to 6.13)	
Non-Hispanic Black	0 [Reference]	−4.23 (−9.10 to 0.65)	5.19 (−0.94 to 11.32)	
Non-Hispanic White	0 [Reference]	−1.68 (−13.21 to 9.85)	−7.90 (−21.69 to 5.90)	
Non-Hispanic Other^d^	0 [Reference]	1.57 (−5.20 to 8.33)	−0.06 (−7.85 to 7.73)	
**Household reported being food secure, PRR (95% CI)^e^^,^ f**				
Hispanic English-speaking	1 [Reference]	1.28 (1.20 to 1.36)	1.17 (1.08 to 1.27)	.15
Hispanic Spanish-speaking	1 [Reference]	1.16 (1.07 to 1.26)	1.12 (1.00 to 1.25)	
Non-Hispanic Asian	1 [Reference]	1.13 (0.92 to 1.39)	1.36 (0.97 to 1.90)	
Non-Hispanic Black	1 [Reference]	1.32 (1.14 to 1.53)	1.07 (0.88 to 1.30)	
Non-Hispanic White	1 [Reference]	1.69 (1.09 to 2.63)	1.42 (0.82 to 2.46)	
Non-Hispanic Other^d^	1 [Reference]	1.20 (0.96 to 1.50)	1.06 (0.83 to 1.35)	
**Child fruit and vegetable intake, difference (95% CI), cups per day^c^^,^ g**				
Hispanic English-speaking	0 [Reference]	−0.08 (−0.15 to −0.02)	−0.17 (−0.25 to −0.09)	.30
Hispanic Spanish-speaking	0 [Reference]	−0.02 (−0.11 to 0.06)	−0.14 (−0.23 to −0.04)	
Non-Hispanic Asian	0 [Reference]	−0.06 (−0.29 to 0.18)	0.14 (−0.14 to 0.43)	
Non-Hispanic Black	0 [Reference]	−0.05 (−0.18 to 0.08)	−0.12 (−0.28 to 0.04)	
Non-Hispanic White	0 [Reference]	0.10 (−0.21 to 0.41)	0.16 (−0.22 to 0.54)	
Non-Hispanic Other^d^	0 [Reference]	0.12 (−0.06 to 0.30)	−0.04 (−0.26 to 0.17)	
**Reported satisfaction with CVB amount, PRR (95% CI)^e^^,^ h**				
Hispanic English-speaking	1 [Reference]	13.66 (10.31 to 18.11)	7.33 (5.50 to 9.78)	.002
Hispanic Spanish-speaking	1 [Reference]	6.48 (5.06 to 8.30)	3.76 (2.89 to 4.88)	
Non-Hispanic Black	1 [Reference]	10.28 (6.50 to 16.27)	7.45 (4.70 to 11.80)	
Non-Hispanic Other^d^	1 [Reference]	6.96 (4.16 to 11.66)	4.12 (2.29 to 7.44)	

Abbreviations: CVB, cash value benefit; PRR, prevalence rate ratio; WIC, Special Supplemental Nutrition Program for Women, Infants, and Children.

a Data source: a prospective cohort study comprising 3 survey waves of caregivers with children aged 1 to 4½ years at baseline who were receiving WIC in 7 WIC clinics in southern California at the following 3 time points: pre-augmentation at $9 per month (Survey 1, baseline, April–May 2021), during the 4-month increase to $35 per month (Survey 2, August–September 2021), and after the CVB was adjusted to $24 per month (Survey 3, April–May 2022) (10). Associations represent the estimated difference from the reference time point (Survey 1) for all outcomes.

b *P* values are for the interaction between race and ethnicity and time point.

c Associations for continuous outcomes (CVB redemption amount, CVB redemption percentage, and fruit and vegetable intake) were evaluated with interaction terms between race and ethnicity and time point in mixed effects regression models accommodating repeated observations of individual children and clustering within families, adjusted for number of children receiving WIC in the household and the presence of ≥3 children (aged <18 y) in the household.

d Includes multiple races, Fijian, Samoan, Tongan, Guamanian or Chamorro, Native Hawaiian, American Indian or Alaska Native.

e Associations for dichotomous outcomes (household food security and satisfaction with CVB amount) were evaluated with interaction terms between race and ethnicity and time point in generalized estimating equation modified Poisson regression models with robust SE estimation, accommodating repeated observations of individual children and clustering within families, adjusted for number of children receiving WIC in the household and the presence of ≥3 children (aged <18 y) in the household.

f Responses to question on food security were dichotomized as food secure or food insecure.

g The model with fruit and vegetable intake was additionally adjusted for baseline fruit and vegetable intake and child sex and included random intercepts and random slope for child age.

h Responses were dichotomized into satisfied (too much, just right) and not satisfied (not enough). Non-Hispanic Asian and non-Hispanic White groups were excluded from this analysis due to small cell sizes.

**Table 4:** Racial and Ethnic Differences in Outcomes, Stratified by Time Point, Among a Sample of Children Participating in WIC, Southern California, 2021–2022^a^

Item	Hispanic		Non-Hispanic				*P* value
	English-speaking (n = 798)	Spanish-speaking (n = 532)	Asian (n = 65)	Black (n = 222)	White (n = 39)	Other^b^ (n = 114)	
**CVB redemption amount, difference (95% CI), in US dollars^c^**							
Survey 1	0 [Reference]^d^	0.30 (−1.19 to 1.78)^d^	−0.25 (−3.58 to 3.08)^e^^,^ f	−0.50 (−2.62 to 1.62)^g^	−1.53 (−5.85 to 2.80)^f^	0.11 (−2.61 to 2.84)^e^	.002
Survey 2	0 [Reference]^f^	−0.25 (−1.85 to 1.35)^f^	−1.30 (−4.94 to 2.34)^e^^,^ g	−4.51 (−6.80 to −2.23)^e^	0.34 (−4.38 to 5.06)^d^^,^ e	2.15 (−0.72 to 5.03)^d^^,^ g	.02
Survey 3	0 [Reference]	0.85 (−1.15 to 2.85)	−2.95 (−7.85 to 1.95)	−1.92 (−4.90 to 1.07)	−2.80 (−9.17 to 3.58)	3.61 (−0.02 to 7.23)	.44
**Percentage of total CVB allotment that was redeemed, difference (95% CI)^c^**							
Survey 1	0 [Reference]^f^	4.47 (1.25 to 7.70)^e^	5.24 (−2.15 to 12.62)^d^	−9.81 (−14.20 to −5.42)^g^	0.66 (−8.73 to 10.06)^d^	−4.89 (−10.67 to 0.89)^d^^,^ g	<.001
Survey 2	0 [Reference]^e^^,^ d	7.00 (3.50 to 10.50)^d^	2.11 (−5.84 to 10.05)^e^^,^ f	−11.21 (−16.00 to −6.42)^g^^,^ h	1.82 (−8.57 to 12.20)^f^^,^ g	−0.49 (−6.81 to 5.83)^f^	<.001
Survey 3	0 [Reference]^d^^,^ f	7.98 (3.67 to 12.30)^d^	0.92 (−9.50 to 11.34)^e^^,^ f	−4.16 (−10.48 to 2.16)^e^^,^ f	−6.77 (−19.71 to 6.18)^e^^,^ g	−4.49 (−12.11 to 3.13)^e^^,^ g	.001
**Household reported being food secure, PRR (95% CI)^i^^,^ j**							
Survey 1	1 [Reference]^d^	0.91 (0.81 to 1.03)^d^^,^ e	0.96 (0.74 to 1.25)^d^^,^ e	0.78 (0.65 to 0.94)^e^	0.66 (0.42 to 1.03)^d^^,^ e	0.86 (0.69 to 1.08)^d^^,^ e	.03
Survey 2	1 [Reference]^d^	0.83 (0.75 to 0.92)^e^	0.85 (0.67 to 1.08)^d^^,^ e	0.81 (0.70 to 0.94)^e^	0.87 (0.64 to 1.19)^d^^,^ e	0.81 (0.66 to 1.00)^e^	.002
Survey 3	1 [Reference]^d^	0.87 (0.77 to 0.99)^d^^,^ e	1.11 (0.84 to 1.47)^d^^,^ e	0.71 (0.58 to 0.88)^e^	0.80 (0.49,1.29)^d^^,^ e	0.78 (0.60 to 1.01)^d^^,^ e	.02
**Child fruit and vegetable intake, difference (95% CI), cups per day^c^^,^ k**							
Survey 1	0 [Reference]	−0.11 (−0.19 to −0.03)	−0.12 (−0.30 to 0.06)	0.01 (−0.10 to 0.11)	−0.13 (−0.36 to 0.10)	−0.08 (−0.22 to 0.06)	.14
Survey 2	0 [Reference]	−0.05 (−0.14 to 0.04)	−0.09 (−0.29 to 0.11)	0.04 (−0.08 to 0.16)	0.05 (−0.21 to 0.31)	0.12 (−0.03 to 0.28)	.22
Survey 3	0 [Reference]	−0.07 (−0.18 to 0.03)	0.19 (−0.07 to 0.45)	0.05 (−0.10 to 0.21)	0.20 (−0.14 to 0.53)	0.04 (−0.16 to 0.24)	.05
**Reported satisfaction with CVB amount, PRR (95% CI)^i^^,^ l**							
Survey 1	1 [Reference]^d^	1.92 (1.32 to 2.79)^e^	—m	1.34 (0.78 to 2.29)^d^^,^ e	—m	1.87 (1.03 to 3.40)^e^	.01
Survey 2	1 [Reference]^e^	0.91 (0.85 to 0.98)^d^	—m	1.01 (0.92 to 1.10)^e^	—m	0.95 (0.83 to 1.09)^d^^,^ e	.03
Survey 3	1 [Reference]	0.98 (0.82 to 1.18)	—m	1.36 (1.11 to 1.67)	—m	1.05 (0.76 to 1.45)	.11

Abbreviations: CVB, cash value benefit; PRR, prevalence rate ratio; WIC, Special Supplemental Nutrition Program for Women, Infants, and Children.

a Data source: a prospective cohort study comprising 3 survey waves of caregivers with children aged 1 to 4½ years at baseline who were receiving WIC in 7 WIC clinics in southern California at the following 3 time points: pre-augmentation at $9 per month (Survey 1, baseline, April–May 2021), during the 4-month increase to $35 per month (Survey 2, August–September 2021), and after the CVB was adjusted to $24 per month (Survey 3, April–May 2022) (10). Associations represent the estimated difference from the reference group, Hispanic English-speakers, for all outcomes.

b Includes multiple races, Fijian, Samoan, Tongan, Guamanian or Chamorro, Native Hawaiian, American Indian or Alaska Native.

c Associations for continuous outcomes (CVB redemption amount, CVB redemption percentage, and fruit and vegetable intake) were evaluated in mixed effects regression models accommodating repeated observations of individual children and clustering within families, adjusted for number of children receiving WIC in the household and the presence of ≥3 children (aged <18 y) in the household.

d,e,f,g,h Values sharing a common superscripted letter are not significantly different from each other in pairwise comparisons that used a .05 level of significance.

i Associations for dichotomous outcomes (household food security and satisfaction with CVB amount) were evaluated in generalized estimating equation modified Poisson regression models with robust standard error estimation, accommodating repeated observations of individual children and clustering within families, adjusted for number of children receiving WIC in the household and the presence of ≥3 children (aged <18 y) in the household.

j Responses to question on food security were dichotomized as food secure or food insecure.

k The model with fruit and vegetable intake was additionally adjusted for baseline fruit and vegetable intake and child sex and included random intercepts and random slope for child age.

l Responses were dichotomized into satisfied (too much, just right) and not satisfied (not enough).

m Non-Hispanic Asian and non-Hispanic White groups were excluded from this analysis due to small cell sizes.

Percentage of CVB redeemed at baseline ranged from 77.0% among non-Hispanic Black respondents to 92.0% among non-Hispanic Asian respondents. We found no significant change in redemption rates for any group throughout the study period (Table 2). However, percentage of CVB redeemed consistently differed between groups (Table 4). Hispanic Spanish-speakers had higher redemption rates than several other groups at all time points.

### Household food security

The prevalence of household food security ranged from 33.3% among White respondents to 48.9% among Hispanic English-speakers (Table 2). Household food security improved from baseline for several groups at Survey 2 and Survey 3, including Hispanic English-speaking, Hispanic Spanish-speaking, and non-Hispanic Black groups. Race and ethnicity was not an effect modifier for change in household food security over time (Table 3). The prevalence of household food security differed significantly between groups at all 3 time points (Table 4). Non-Hispanic Black households had a lower prevalence than Hispanic English-speaking households at all 3 time points, and Hispanic Spanish-speakers and non-Hispanic Other households had a lower prevalence than Hispanic English-speaking households at Survey 2.

### Child fruit and vegetable intake

Mean fruit and vegetable intake at baseline ranged from 2.6 cups per day among Hispanic English-speakers to 2.3 cups per day among non-Hispanic White respondents (Table 2). From baseline to Survey 3, Hispanic English-speaking and Hispanic Spanish-speaking groups reported a decrease in fruit and vegetable intake. For all other groups, we observed no significant associations across time points. Group was not an effect modifier (Table 3). For all time points, fruit and vegetable intake was not significantly different between racial and ethnic groups (Table 4).

### Satisfaction with CVB amount

Baseline satisfaction with the $9 CVB ranged from 2.6% among non-Hispanic White respondents to 11.0% among Hispanic Spanish-speakers (Table 2). Satisfaction increased among all groups at both follow-up time points compared with baseline; satisfaction rates were highest in Survey 2. Changes in satisfaction differed significantly by racial and ethnic group (Table 3). At both follow-up time points, increases in satisfaction were larger among Hispanic English-speakers and non-Hispanic Black respondents than among Hispanic Spanish-speakers and the non-Hispanic Other group, who started with higher baseline values. At baseline, satisfaction was higher among Hispanic Spanish-speakers (PRR = 1.92, 95% CI, 1.32–2.79) and the non-Hispanic Other group (PRR = 1.87; 95% CI, 1.03–3.40) than among Hispanic English-speakers (Table 4). At Survey 2, satisfaction rates among Hispanic English-speaking and non-Hispanic Black groups (PRR = 1.01; 95% CI, 0.92–1.10) surpassed the rate among Hispanic Spanish-speakers (PRR = 0.91; 95% CI, 0.85–0.98). At Survey 3, satisfaction rates were similar across groups. Although the non-Hispanic Asian and non-Hispanic White groups were excluded from this analysis because of small sample sizes, from a descriptive standpoint, the 2 groups started with low rates of satisfaction and saw large increases, with 3.1% and 2.6% at baseline, then increasing to 66.7% and 69.2% at Survey 2, and 38.6% and 33.3% at Survey 3, respectively.

### Survey 3 descriptive analyses

At Survey 3, a majority in each group reported that the variety and amount of fruits and vegetables consumed by their eldest child receiving WIC had increased from when the CVB was $9 per month (Table 5). The changes in variety of fruits and vegetables and amount of fruits consumed did not differ across groups. The change in the amount of vegetables consumed differed significantly among racial and ethnic groups; the percentage reporting an increase was significantly smaller among Hispanic English-speakers (60.1%) than among Hispanic Spanish-speakers (74.5%) and non-Hispanic Other respondents (78.7%). The likelihood of continuing with WIC if the CVB returned to $9 per month differed significantly across groups. Hispanic Spanish-speakers reported a higher likelihood of staying on the program (90.0%) than Hispanic English-speakers (75.2%), non-Hispanic Asian (62.5%), non-Hispanic Black (73.3%), and non-Hispanic Other (74.2%) respondents.

**Table 5:** Perception of Changes in Variety and Amount of Fruits and Vegetables Consumed by Child After Cash Value Benefit Was Augmented to $24 per Month, and Likelihood of Continued WIC Participation Among a Sample of Children Participating in WIC, Southern California, 2022^a^

Item	Hispanic		Non-Hispanic				*P* value^c^
	English-speaking (n = 408)	Spanish-speaking (n = 301)	Asian (n = 32)	Black (n = 105)	White (n = 19)	Other^b^ (n = 62)	
Increased variety of fruits	337 (83.4)	256 (87.4)	29 (90.6)	86 (84.3)	18 (94.7)	53 (88.3)	.28
Increased amount of fruits	311 (77.9)	240 (83.0)	25 (80.7)	84 (82.4)	14 (73.7)	49 (81.7)	.62
Increased variety of vegetables	270 (68.5)	223 (76.4)	23 (74.2)	74 (72.6)	16 (84.2)	44 (73.3)	.21
Increased amount of vegetables	241 (60.1)^d^	216 (74.5)^e^	22 (71.0)^d^^,^ e	71 (69.6)^d^^,^ e	12 (63.2)^d^^,^ e	48 (78.7)^d^	.001
Likely to come back to WIC if cash value benefit went back to $9	306 (75.2)^e^	271 (90.0)^e^	20 (62.5)^e^	77 (73.3)^e^	13 (68.4)^e^	46 (74.2)^e^	<.001

Abbreviation: WIC, Special Supplemental Nutrition Program for Women, Infants, and Children.

a Data source: a prospective cohort study comprising 3 survey waves of caregivers with children aged 1 to 4½ years at baseline who were receiving WIC in 7 WIC clinics in southern California at the following 3 time points: pre-augmentation at $9 per month (Survey 1, baseline, April–May 2021), during the 4-month increase to $35 per month (Survey 2, August–September 2021), and after the CVB was adjusted to $24 per month (Survey 3, April–May 2022) (10). All responses are from Survey 3. All values are number (percentage) unless indicated otherwise; total sample size may vary because of nonresponse.

b Includes multiple races, Fijian, Samoan, Tongan, Guamanian or Chamorro, Native Hawaiian, American Indian or Alaska Native.

c Differences in frequency by race and ethnicity were tested by using generalized estimating equation modified Poisson regression models with robust SE estimation, accommodating repeated observations of individual children and clustering within families adjusted for number of children receiving WIC in the household and the presence of ≥3 children (aged <18 y) in the household.

d,e Values sharing a common superscripted letter are not significantly different from each other in pairwise comparisons that used a .05 level of significance.

## Discussion

Our study on the CVB augmentation in WIC in 2021 and 2022 identified its benefits among racial and ethnic groups. We observed the largest changes in the amount of CVB redeemed, food security, and satisfaction with the CVB amount for most groups at the $35-per-month level compared with the $9-per-month level; however, the $24-per-month benefit was associated with substantially better outcomes than the original $9 per month. Both CVB amount redeemed and household food security increased from baseline to follow-up, although disparities in household food security among non-Hispanic Black respondents persisted, indicating the need for interventions beyond CVB augmentation. Nonetheless, families faced hardships during the COVID-19 pandemic, and our study, along with other qualitative studies, demonstrated a need for increases in the CVB (14,30). Additionally, although the dollar amounts of CVB redeemed increased among all groups, we found that racial and ethnic groups varied in their percentage of CVB redeemed at each time point. Our findings were consistent with previous reports on racial and ethnic differences in WIC food package redemption, which found higher redemption percentages among Hispanic Spanish-speakers (31). While further research is needed on factors driving these differences, insufficient supply of WIC-eligible items in stores and access to WIC-approved vendors can be barriers to redemption (14). Future studies should explore how barriers and retail environments are experienced differently by racial and ethnic groups and examine their effects on CVB redemption. WIC clinics are also well positioned to provide culturally tailored nutrition counseling that features CVB-eligible items, which may influence use of CVB.

Our study also found substantial increases in satisfaction with the CVB, which differed across groups. Satisfaction is a critical indicator of retention in WIC — low satisfaction with the WIC food package is commonly cited as a reason for leaving the program (32). The substantial increases in satisfaction suggest that the augmented CVB was particularly well-received among non-Hispanic Asian, non-Hispanic Black, and non-Hispanic White respondents. These groups also reported that they would be less likely to continue with WIC if the CVB returned to $9. Taken together, our results suggest that CVB value may strongly influence the decision among people in these groups to participate in WIC. Nationally, these groups generally have lower WIC participation rates (33). Their lower participation rates may be due to perceived inadequacy of culturally appropriate foods in the WIC food package (16). Our results highlight the importance of examining the influence of the CVB on WIC participation among racial and ethnic groups and on reducing health disparities (33).

An unexpected result of our study was that fruit and vegetable intake did not increase across any racial and ethnic group. Notably, the lowest average intake among children in our sample (2.33 cups per day in the non-Hispanic White group) before CVB augmentation was higher than the average intake among all children (2.31 cups per day) in another, multistate study after CVB augmentation; participants in that study increased their intake by ⅓ cup (11). Because the recommended daily fruit and vegetable intake for WIC-aged children is approximately 2½ cups, it may be difficult to document increases in fruit and vegetable intake in a population that is already consuming relatively high levels (21). Results of a previous analysis found that children with the lowest baseline fruit and vegetable intake experienced significant increases in fruit and vegetable intake, indicating that benefits are likely reaching those with the greatest need (10). Results from Survey 3 indicated that, for most respondents, the CVB augmentation increased the variety and amount of fruits and vegetables consumed. The CVB augmentation may have allowed parents to offer a larger quantity or a wider variety of produce that included more expensive items (eg, berries in addition to apples), as supported by a study that used purchasing data from WIC participants (12,34).

### Limitations

This study has several limitations. Participants were limited to English- and Spanish-speakers, and many non-Hispanic Asian and non-Hispanic White WIC participants in our study area prefer a language other than English or Spanish, limiting representativeness of the results. Among Los Angeles County WIC families in May 2021, 60% of Asian families and 20% of non-Hispanic White families preferred a language other than English or Spanish (35). The demographic characteristics of WIC participants in southern California may limit generalizability to other populations, reflected in the relatively small sample of non-Hispanic White participants. Furthermore, a small sample size in some racial and ethnic groups may have reduced our ability to detect statistical differences. Because the study sample consisted of participants who were willing to respond to texts and complete online surveys, there may be nonresponse bias, overrepresenting those with technology access. Finally, because we did not prespecify hypotheses on differences between racial and ethnic groups, our study was exploratory.

### Conclusion

Augmentation of the CVB in the WIC program in 2021 and 2022 was associated with numerous benefits, including increases in redemption, food security, and satisfaction. It presents a promising strategy to increase the well-being of WIC participants in all racial and ethnic groups. In allowing participants the flexibility to select fruits and vegetables that meet their household and cultural preferences, continued investment in an augmented CVB will allow WIC to serve its diverse population and can improve health outcomes. Future research that examines differences among racial and ethnic groups in factors influencing CVB redemption, whether the augmented CVB increased the uptake and retention of WIC across groups, and how the CVB affects fruit and vegetable purchasing is needed.
